# Measuring Habitual Arm Use Post-stroke With a Bilateral Time-Constrained Reaching Task

**DOI:** 10.3389/fneur.2018.00883

**Published:** 2018-10-22

**Authors:** Sujin Kim, Hyeshin Park, Cheol E. Han, Carolee J. Winstein, Nicolas Schweighofer

**Affiliations:** ^1^Biokinesiology and Physical Therapy, University of Southern California, Los Angeles, CA, United States; ^2^Physical Therapy, Jeonju University, Jeonju, South Korea; ^3^Department of Neuroscience, Baylor College of Medicine, Houston, TX, United States; ^4^Department of Electronics and Information Engineering, Korea University, Sejong, South Korea

**Keywords:** stroke, hemiparesis, arm use, habitual choice, decision making

## Abstract

**Background:** Spontaneous use of the more-affected arm is a meaningful indicator of stroke recovery. The Bilateral Arm Reaching Test (BART) was previously developed to quantify arm use by measuring arm choice to targets projected over a horizontal hemi-workspace. In order to improve clinical validity, we constrained the available movement time, thereby promoting more spontaneous decision making when selecting between the more-affected and less affected arm during the BART.

**Methods:** Twenty-two individuals with mild to moderate hemiparesis were tested with the time-based BART in three time-constraint conditions: no-time constraint, medium, and fast conditions. Arm use was measured across three sessions with a 2-week interval in a spontaneous choice block, in which participants were instructed to use either the more-affected or the less-affected arm to reach targets. We tested the effect of time-constraint condition on the more-affected arm use, external validity of the BART with the Actual Amount of Use Test (AAUT), and test-retest reliability across the three test sessions.

**Results:** The fast condition in the time-based BART showed reduced use of the more-affected arm compared to the no-time constraint condition *P* < 0.0001) and the medium condition *P* = 0.0006; Tukey *post hoc* analysis after mixed-effect linear regression). In addition, the fast condition showed strong correlation with the AAUT *r* = 0.829, *P* < 0.001), and excellent test-retest reliability (ICC = 0.960, *P* < 0.0001).

**Conclusion:** The revised BART with a time-restricted fast condition provides an objective, accurate, and repeatable measure of spontaneous arm use in individuals with chronic stroke hemiparesis.

## Introduction

Spontaneous use of the more-affected upper extremity post-stroke is often lower than would be expected from impairment levels (1, 2), with low use associated with a reduced quality of life (3). Besides the common therapy goal of improving motor performance of the more-affected arm/hand, an additional approach would be to influence the decision-making system (4), with the aim to improve use of the more-affected arm/hand.

The three instruments commonly used for measuring spontaneous arm/hand use in the natural environment are the Motor Activity Log [MAL; (5)], the Actual Amount of Use Test [AAUT; (6)], and accelerometers (7, 8). These instruments are not ideal, however: the MAL relies on self-reported ratings from memory; the AAUT cannot be administered repeatedly once participants recognize that they are being tested, thereby revealing its covert nature; and accelerometers only provide overall activity, and thus not a direct measure of functional arm use.

We previously developed a simple and objective assessment tool, the Bilateral Arm Reaching Test (BART) to address these limitations (1). With BART, arm use is measured in a *spontaneous choice block*, in which participants are instructed to choose either the more-affected or the less-affected arm to reach displayed targets on a table. Although arm use as assessed with BART showed good test-retest reliability, it was only moderately correlated with the AAUT (1). In seeking to improve BART, we sought a better way to capture real-world spontaneous arm use. We turned to previous research in decision-making (9–11). Contemporary decision models posit that choices between potentially rewarding actions are driven by a combination of a goal-oriented system and a habitual system. The goal-directed system is called “model-based” because individuals learn through experience, and then mentally simulate, models of the decision environment to prospectively evaluate the outcomes of possible actions. In contrast, the habitual system is “model-free,” because choice is performed via direct comparison of expected rewards for each potential action (12). Mental simulations in the goal-directed system is a time-consuming process. As a result, performing choices under time-pressure enhances expression of the time-insensitive habitual system (13). For this reason, we modified BART by adding a short time-constraint condition to the experimental paradigm.

The aim of this study was to accurately quantify arm/hand use post-stroke with the time-based BART system. We hypothesized that a reduction of available decision time would reduce affected arm use. In addition, we reasoned that affected arm use in the time-constrained condition would more strongly correlate with arm use as assessed by the covert AAUT than arm use without time constraint.

## Materials and methods

### Participants

Twenty-two right-handed stroke participants with chronic stroke and mild to moderate upper extremity impairments were recruited as part of a sub-cohort of the DOSE phase 1 randomized controlled trial (NCT 01749358). Here, we only included baseline BART data, that is, data obtained before the DOSE intervention. Inclusion criteria were: (1) ischemic or intraparenchymal hemorrhagic stroke without intraventricular extension with confirmatory neuroimaging more than 180 days (6 months) after onset; (2) Age ≥21 and no upper limit; (3) impaired arm/hand motor function indicated by the Fugl-Meyer motor and coordination score no less than 19 out of 66 on the total motor score (14); (4) no arm/hand neglect as determined by Albert Test; (5) Mini-Mental State Examination (MMSE) score >24/30; and (6) no previous or current musculoskeletal injury or conditions that limited arm/hand use. We excluded participants if they were left-handed or could not reach the farthest straight-ahead target in the BART display (30 cm away from the home position; see below). The study was approved by the Human Research and Review Committee of the University of Southern California and each participant signed an informed consent.

### Experimental setup and task

**Figure 1:**
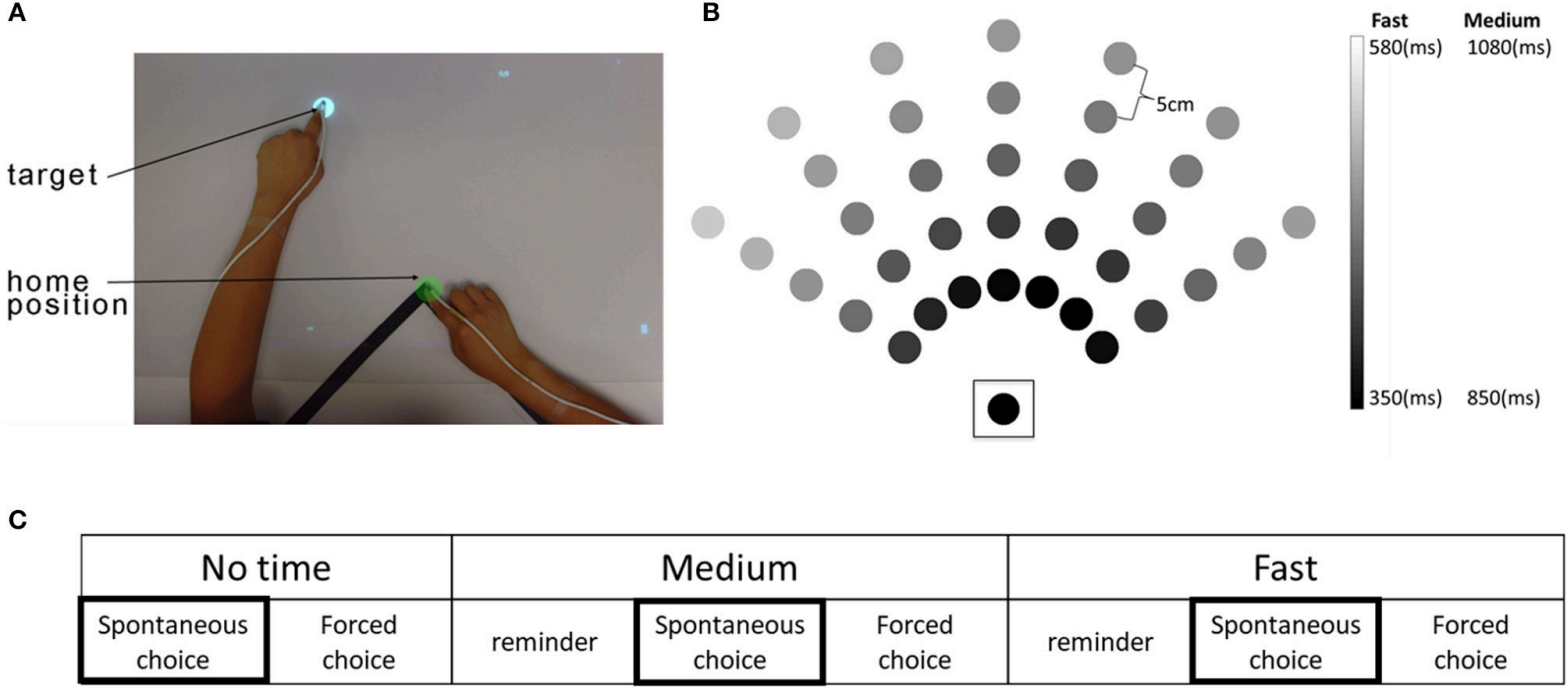
The time-based Bilateral Arm Reaching Test (BART), target location, and experimental protocol. **(A)** Participants sat in a chair with a trunk-restraining belt. A magnetic sensor attached to the tips of each index finger. Home position and target are shown in green and white colors, respectively. **(B)** BART workspace with 35 targets (the home position is enclosed by a square). Movement duration constraints for each target varied as a function of target distance and angle. Shading shows the time constraints for each target in the medium and the fast conditions. **(C)** The experimental protocol. Each condition was presented in a spontaneous choice (participant chooses the arm/hand for the reach) and forced choice (investigator determines the arm/hand for the reach) block. A reminder block was given before the spontaneous choice block for both medium and fast conditions. Arm use/choice in the three spontaneous choice blocks was used for data analysis.

The time-based BART system consists of a computer, an over-head projector illuminating virtual targets on a table surface, two magnetic sensors placed on the index finger of each hand, and a seat belt to prevent compensational trunk movements during reaching (Figure 1A). The detailed physical set up is described in our previous study (1). At each trial, a virtual target (white disk, 2 cm in diameter) appeared at one of 35 possible target locations (Figure 1B). There were three movement duration conditions with three levels of time constraint: a no-time constraint, a medium time constraint, and a fast time constraint condition (Figure 1C). Whereas in the no time constraint condition, targets did not disappear until they were captured, in the fast and medium conditions, targets disappeared after movement onset following condition-dependent and target-dependent time constraints: 350–580 ms in the fast condition and 500 ms longer for all targets (i.e., 850–1,080 ms) in the medium condition. The target-dependent time constraints, which were estimated using previous reaching data from non-disabled participants, account for longer movement times for far away targets and for targets that require coordinated elbow and shoulder movements [see Figure 1B and (15)].

Each time-constraint condition consisted of a spontaneous choice block to measure spontaneous arm use and a forced choice block to measure performance of the investigator-specified limb (Figure 1C). Here, we only report results from the spontaneous choice blocks, which was always given before the forced choice blocks to prevent bias in hand use. In the spontaneous choice block, participants were free to choose either the more-affected or the less-affected arm to reach each target, with two trials per target (i.e., 70 trials per block). In each spontaneous choice block condition, we measured use in by counting the number of targets successfully captured using the more-affected arm, within the time constraint.

For the medium and fast speed conditions, a reminder block (similar to the spontaneous choice block, 35 trials per block) was provided before the spontaneous choice block (Figure 1C). Participants were asked to reach the targets as rapidly and accurately as possible throughout all conditions and blocks. Participants performed three BART sessions, with a 2-week interval between sessions.

### Clinical assessments

We used the Actual Amount of Use Test (AAUT), specifically, the AAUT quality of movement scale (QOM) to assess spontaneous use of the arm/hand (6, 16). From a videotaped record acquired without the participants' awareness (i.e., covert administration), the trained and standardized evaluator scored the participants' spontaneous arm use behavior during 14 upper-extremity daily tasks, such as opening a file folder, and writing on and folding up a piece of paper. The QOM score for each item was averaged over the 14 tasks (6).

### Statistical analysis

The effect of the time constraint on arm use was analyzed using mixed effect models with condition (no time-, medium-, and fast-constraint) as fixed factors and participants as a random factor. *Post-hoc* analyses were performed using Tukey's test, which corrects for multiple comparisons. External validity for the no time, medium, and fast conditions, for the third test session was tested using correlations with the AAUT QOM. Test-retest reliability for the time-based BART was assessed using intraclass correlation coefficients (ICCs) for the three test sessions (test 1, 2, and 3). Significance threshold was set at *P* = 0.05, and statistics were run using customized code in R and MATLAB. All results are reported as average ± SEs.

## Results

**Figure 2:**
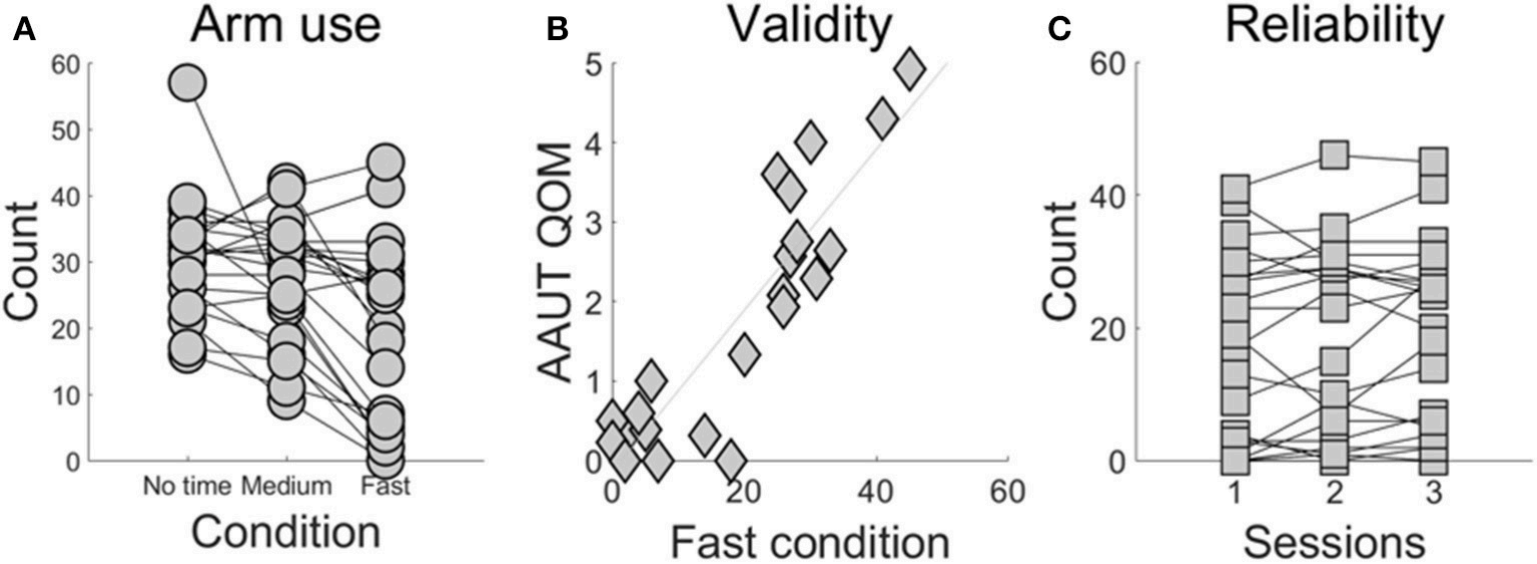
BART assessment of use as a function time constraint condition **(A)**, external validity of the fast condition **(B)**, and test-retest reliability of the fast condition **(C)**. **(A)** Use of the more-affected arm in the fast condition is lower than use in the no time constraint and the medium condition (*P* < 0.001). Each line represents a participant post-stroke tested in the three conditions and each dot represents the number of targets that each subject successfully reached using the more-affected arm. **(B)** Arm use scores the fast condition and from the AAUT QOM show excellent correlation (*r* = 0.829, *P* < 0.001). **(C)** The fast condition shows excellent test-retest reliability (ICC = 0.960, *P* < 0.0001).

Demographic information and stroke-specific characteristics are provided in Supplementary Table 1. Figure 2A shows the number of times the more-affected arm was successfully used across the three conditions. More-affected arm use decreased in the fast- (18.9 ± 2.9) compared to the medium- (27.5 ± 1.9) and compared to the no time constraint-condition (30.7 ± 1.8; *P* < 0.0001 between the fast- and no time constraint-condition, *P* = 0.0006 between the fast- and medium-condition).

Arm use computed in the fast condition was strongly correlated with AAUT QOM use score (*r* = 0.829, *P* < 0.001, Pearson correlation; Figure 2B). In contrast, arm use in the medium condition showed a moderately strong correlation with AAUT QOM (*r* = 0.538, *P* = 0.009, Spearman correlation), and arm use in the no-time constraint condition showed no significant correlation (*r* = 0.363, *P* = 0.096, Spearman correlation).

The fast condition showed excellent test-retest reliability (ICC = 0.960, *P* < 0.0001; Figure 2C). In contrast, the medium- and no time constraint conditions showed lower reliability (ICC for the medium condition: 0.815, *P* < 0.0001 and no time constraint condition: 0.691, *P* < 0.0001).

## Discussion

Our primary aim was to objectively quantify use of the more-affected arm during targeted reaching movements using a novel, theoretically-motivated, temporally-constrained version of BART in individuals post-stroke. Our results demonstrate that individuals in the chronic stage post-stroke with mild to moderate hemiparesis decrease their use of the more-affected arm in the fast condition, compared to the other two, less time constrained (no-time- and medium time constraint), conditions. In addition, and in support of our hypothesis, we found a strong and significant correlation between arm use measured in the fast time-constrained condition and arm use assessed with an often-used clinical tool, the AAUT QOM. Thus, the time-based BART appears to reflect an accurate assessment of real-world arm use. Compared to the original BART, this time-based BART may represent a more ecologically valid measure of arm choice/use, because it nudges the participant into decision-making under time pressure; a situation previously shown to enhance the expression of the habitual choice system (13).

Arm choice is a flexible and dynamic process that depends on the task environment. Previous research with non-disabled participants shows that arm choice is modulated by the task demands. For instances, (i) introduction of an abrupt force on one hand quickly reduces the choice of that hand for action (17), (ii) a reduction in target size leads to a reduced choice of the non-dominant hand (18), and (iii) a decreased success rate for one hand yields reduced choice of that hand (19). Additionally, individuals who are recovering from a stroke use their more-affected arm less as tasks became more challenging (20). In the time-based BART, the time constraint puts pressure on movement time as well as decision time. It is known that faster reaching is more challenging than reaching at preferred speed (21). Our results therefore indicate that, under time constraint, individuals with stroke decrease use of their more affected arm to maximize success with the task.

Thus, the time-based BART appears to be a viable alternative to the AAUT, because it captures use of the arm/hand objectively and repeatedly in chronic stroke survivors with mild to moderate arm/hand motor impairment. In addition, BART is easy to administer and requires minimal training.

However, additional testing is needed before the time-based BART can be used to replace the AAUT in the general stroke population for three reasons. First, we included a relatively small number of individuals chronically post-stroke, specifically, those with mild to moderate motor impairments. Second, because of the difficulty in recruiting pre-stroke left-hand dominant participants, we only included right-hand dominant individuals. Finally, because the no time constraint condition was presented first, participants may have accumulated fatigue by the time they experienced the fast condition (22). Thus, we cannot rule out the possibility that fatigue may have influenced use/choice of the more-affected arm in the fast condition. Nevertheless, we chose to start with the no time constraint condition in order to prevent “zero-use” of the more-affected arm in the fast condition, something we observed in our pilot studies, for some participants, regardless of capability to reach targets successfully.

Finally, given that the time-based BART assesses aiming movement, whereas the AAUT assesses both arm and hand movements that involve grasp manipulation or stabilization, and bi-manual tasks, one may question the validity findings. We offer four possible explanations: (i) the fast condition in the time-based BART provides an accurate expression of the habitual system for arm choice, in large part due to the time pressure which prevents full engagement of the goal-oriented system, (ii) the AAUT, by its covert nature, captures habitual and spontaneous use of the more-affected arm, (iii) both the BART and AAUT evaluate the speed and accuracy of the more-affected arm, (iv) the habitual system for arm choice is not well tuned to the specific task requirements. In contrast, the goal-oriented choice system would be, via simulation of the motor system, well-tuned to specific motor actions. Further work is needed to formally test these possibilities.

## Author contributions

SK designed the study, piloted the study, ran the study, analyzed the data, wrote the manuscript; HP adapted the computer code, piloted the study; CH wrote the initial computer code, provided advice to the design; CW designed the study, wrote the manuscript; NS designed the study, wrote the manuscript.

### Conflict of interest statement

The authors declare that the research was conducted in the absence of any commercial or financial relationships that could be construed as a potential conflict of interest.

## References

[1] Han CE Kim S Chen S Lai YH Lee JY Osu R. Quantifying arm nonuse in individuals poststroke. Neurorehabil Neural Repair (2013) 27:439–47. 10.1177/1545968312471904 23353185 PMC3922644

[2] Sterr A Freivogel S Schmalohr D. Neurobehavioral aspects of recovery: assessment of the learned nonuse phenomenon in hemiparetic adolescents. Arch Phys Med Rehabil. (2002) 83:1726–31. 10.1053/apmr.2002.35660 12474177

[3] Haaland KY Mutha PK Rinehart JK Daniels M Cushnyr B Adair JC. Relationship between arm usage and instrumental activities of daily living after unilateral stroke. Arch Phys Med Rehabil. (2012) 93:1957–62. 10.1016/j.apmr.2012.05.011 22634230

[4] Han CE Arbib MA Schweighofer N. Stroke rehabilitation reaches a threshold. PLoS Comput Biol. (2008) 4:e1000133. 10.1371/journal.pcbi.1000133 18769588 PMC2527783

[5] Uswatte G Taub E Morris D Light K Thompson PA. The motor activity Log-28: assessing daily use of the hemiparetic arm after stroke. Neurology (2006) 67:1189–94. 10.1212/01.wnl.0000238164.90657.c2 17030751

[6] Taub E Crago JE Uswatte G Constraint-induced movement therapy: a new approach to treatment in physical rehabilitation. Rehabil Psychol. (1998) 43:152–70. 10.1037//0090-5550.43.2.152

[7] Bailey RR Klaesner JW Lang CE. Quantifying real-world upper-limb activity in nondisabled adults and adults with chronic stroke. Neurorehabil Neural Repair (2015) 29:969–78. 10.1177/1545968315583720 25896988 PMC4615281

[8] Uswatte G Foo WL Olmstead H Lopez K Holand A Simms LB. Ambulatory monitoring of arm movement using accelerometry: an objective measure of upper-extremity rehabilitation in persons with chronic stroke. Arch Phys Med Rehabil. (2005) 86:1498–501. 10.1016/j.apmr.2005.01.010 16003690

[9] Daw ND Niv Y Dayan P. Uncertainty-based competition between prefrontal and dorsolateral striatal systems for behavioral control. Nat Neurosci. (2005) 8:1704–11. 10.1038/nn1560 16286932

[10] Dolan RJ Dayan P. Goals and habits in the brain. Neuron (2013) 80:312–25. 10.1016/j.neuron.2013.09.007 24139036 PMC3807793

[11] Doya K. What are the computations of the cerebellum, the basal gangila, and the cerebral cortex? Sci Technol. (1999) 12:1–48. 10.1016/S0893-6080(99)00046-5 12662639

[12] Daw ND Gershman SJ Seymour B Dayan P Dolan RJ. Model-based influences on humans' choices and striatal prediction errors. Neuron (2011) 69:1204–15. 10.1016/j.neuron.2011.02.027 21435563 PMC3077926

[13] Keramati M Smittenaar P Dolan RJ Dayan P. Adaptive integration of habits into depth-limited planning defines a habitual-goal–directed spectrum. Proc Natl Acad Sci. (2016) 113:12868–73. 10.1073/pnas.1609094113 27791110 PMC5111694

[14] Fugl-Meyer AR Jääskö L Leyman I Olsson S Steglind S The post-stroke hemiplegic patient. 1. a method for evaluation of physical performance. Scand J Rehabil Med. (1975) 7:13–31. 1135616

[15] Park H Kim S Winstein CJ Gordon J Schweighofer N. Short-duration and intensive training improves long-term reaching performance in individuals with chronic stroke. Neurorehabil Neural Repair (2015)2015:1545968315606990. 10.1177/1545968315606990 26405046 PMC4808509

[16] Uswatte G Giuliani C Winstein C Zeringue A Hobbs L Wolf SL. Validity of accelerometry for monitoring real-world arm activity in patients with subacute stroke: evidence from the extremity constraint-induced therapy evaluation trial. Arch Phys Med Rehabil. (2006) 87:1340–5. 10.1016/j.apmr.2006.06.006 17023243

[17] Habagishi C Kasuga S Otaka Y Liu M Ushiba J. Different strategy of hand choice after learning of constant and incremental dynamical perturbation in arm reaching. Front Hum Neurosci. (2014) 8:92. 10.3389/fnhum.2014.00092 24605097 PMC3932483

[18] Schweighofer N Xiao Y Kim S Yoshioka T Gordon J Osu R. Effort, success, and nonuse determine arm choice. J Neurophysiol. (2015) 114:551–9. 10.1152/jn.00593.2014 25948869 PMC4509397

[19] Stoloff RH Taylor JA Xu J Ridderikhoff A Ivry RB. Effect of reinforcement history on hand choice in an unconstrained reaching task. Front Neurosci. (2011) 5:41. 10.3389/fnins.2011.00041 21472031 PMC3066466

[20] Brown E Hand Preference After Stroke: the Development and Initial Evaluation of a New Performance-Based Measure. Thesis, Dep Kinesiol Univ Waterloo (2011)

[21] Mandon L Boudarham J Robertson J Bensmail D Roche N Roby-Brami A. Faster reaching in chronic spastic stroke patients comes at the expense of arm-trunk coordination. Neurorehabil Neural Repair (2016) 30:209–20. 10.1177/1545968315591704 26089311

[22] Park H Schweighofer N. Nonlinear mixed-effects model reveals a distinction between learning and performance in intensive reach training post-stroke. J Neuroeng Rehabil. (2017) 14:21. 10.1186/s12984-017-0233-2 28302158 PMC5356348

