# Supplementary material for: Measuring Habitual Arm Use Post-stroke With a Bilateral Time-Constrained Reaching Task
Source: Front Neurol. 2018 Oct 22;9:883. doi: 10.3389/fneur.2018.00883 (PMC6213443; doi:10.3389/fneur.2018.00883)
Supplement: Supplementary file 1 [file Table_1.DOCX]

Supplementary Table 1. Demographic information of participants with stroke.

| Subject | Age  (years) | Gender | Stroke Duration (years) | Dominant Hand | More- affected  side | FM |
| --- | --- | --- | --- | --- | --- | --- |
| S1 | 46 | Female | 1.41 | Right | Left | 57 |
| S2 | 62 | Male | 1.39 | Right | Right | 52 |
| S3 | 63 | Male | 5.69 | Right | Right | 53 |
| S4 | 45 | Male | 1.21 | Right | Right | 38 |
| S5 | 56 | Female | 2.10 | Right | Right | 58 |
| S6 | 69 | Male | 0.64 | Right | Right | 53 |
| S7 | 69 | Male | 14.38 | Right | Left | 40 |
| S8 | 35 | Female | 0.53 | Right | Left | 47 |
| S9 | 72 | Male | 4.06 | Right | Left | 51 |
| S10 | 42 | Male | 0.51 | Right | Left | 28 |
| S11 | 40 | Male | 0.55 | Right | Right | 49 |
| S12 | 60 | Female | 0.47 | Right | Left | 36 |
| S13 | 58 | Female | 0.47 | Right | Left | 35 |
| S14 | 61 | Male | 0.98 | Right | Right | 34 |
| S15 | 47 | Male | 1.94 | Right | Left | 28 |
| S16 | 71 | Male | 9.90 | Right | Left | 39 |
| S17 | 76 | Male | 4.00 | Right | Right | 19 |
| S18 | 78 | Male | 1.19 | Right | Right | 50 |
| S19 | 46 | Male | 2.08 | Right | Right | 43 |
| S20 | 78 | Male | 1.76 | Right | Right | 37 |
| S21 | 80 | Male | 4.62 | Right | Right | 53 |
| S22 | 69 | Male | 0.66 | Right | Left | 55 |
| Mean± SE | 60.14±2.94 | 17 Males/  5 Females | 2.75±0.76 | 22 Right | 12Right/  10 Left | 43.41±2.29 |

Abbreviations: SE: standard error, FM: Fugl-Meyer.
